# Risk Factors for Recurrent Fractures in Hip Fracture Patients: A Big Data Analysis of Demographic, Clinical, and Functional Characteristics

**DOI:** 10.3390/jcm14238495

**Published:** 2025-11-30

**Authors:** Avital Hershkovitz, Gal Maydan, Natalia Kornyukov, Yarden Itzhaky

**Affiliations:** 1 Beit Rivka Geriatric Rehabilitation Center, 4 Hachamisha Str., Petach Tikva 4924577, Israel; gald2@clalit.org.il (G.M.); natalyako1@clalit.org.il (N.K.); 2 Gray Faculty of Medical and Health Sciences, Tel-Aviv University, Tel Aviv 6997801, Israel; 3 Data Research Center for Mental Health and Rehabilitation, Clalit Health Services, Petach Tikva 4910002, Israel; yitzhaky@clalit.org.il

**Keywords:** hip fracture, older people, rehabilitation, outcomes, recurrent fracture

## Abstract

**Background/Objectives:** Individuals who have sustained a hip fracture are at an increased risk of experiencing a recurrent fracture. The issue of recurrent fractures in post-acute settings has been scarcely studied. Our aim was to identify independent predictors associated with fracture recurrence. **Methods:** Data were extracted from the Clalit Health Services Research Data Sharing Platform, powered by MDClone. Chi-square and t-tests compared categorical and continuous variables between the two patient groups. Logistic regression analysis was used to identify independent predictors of recurrent fractures following hip fracture. **Results:** The study population comprised 40,391 Clalit Health Services insured patients aged ≥ 65 years who had sustained a hip fracture between the years 2016 and 2023; 23,027 (57%) sustained a single hip fracture during the study period; 17,364 (43%) experienced a recurrent fracture. Independent predictors of recurrent fractures included a documented diagnosis of osteoporosis at the time of the first fracture and a history of cerebrovascular disease, depression, and female gender. **Conclusions:** These findings underscore the multifactorial nature of recurrent fracture risk in older adults following an initial hip fracture. Proactive identification of patients with these risk factors and the implementation of targeted secondary prevention strategies may reduce the incidence of subsequent fractures.

## 1. Introduction

Hip fractures are associated with substantial functional disability, elevated mortality rates in older adults, and a marked reduction in the quality of life [1,2]. Decreased bone mineral density is the most prominent risk factor for an initial hip fracture, accounting for ~60% to 85% of the variance in fracture susceptibility. Additional contributing factors include advanced age, a history of previous fractures, cognitive impairment, functional decline, visual deficits, and unintentional weight loss [3,4].

Osteoporosis is a common disease in older people, albeit, varied among countries and regions [5]. Hip fractures in elderly patients are associated with high morbidity and mortality. Advanced age, comorbidities, and nutritional deficiencies are known prognostic factors for poor outcomes following surgery. A recent study found that the elevated neutrophil-to-lymphocyte ratio (NLR) and platelet-to-lymphocyte ratio (PLR) at admission, are also independent, strong predictors of adverse outcomes in hip fracture elderly patients [6]. Individuals who have sustained a hip fracture are at an increased risk of experiencing a contralateral hip fracture. The incidence of a second hip fracture within the first year following the initial event ranges from 2% to 10%, with lifetime risk estimates approaching 20%. This rate is expected to increase due to the aging population [7]. Evidence from multiple randomized controlled trials and cohort studies have identified advanced age, cognitive impairment, and low bone mass as major predictors of recurrent fractures. Additional risk factors associated with an increased likelihood of a second fracture include impaired depth perception, impaired mobility, a history of recurrent falls, dizziness, Parkinson’s disease, cardiopulmonary comorbidities, and a negative self-perception of health [8,9,10,11,12,13,14].

Several studies have shown that pharmacological treatment for osteoporosis can reduce the risk of subsequent fractures [15,16]. However, other investigations have reported no significant association between osteoporosis treatment and a decreased risk of fracture recurrence [17]. A substantial proportion of patients who sustained a second hip fracture did not receive any form of osteoporosis treatment [18,19]. Patients who sustain a second hip fracture have higher mortality rates compared to those with a single fracture. Furthermore, these patients exhibit lower rates of mobility recovery and a reduced likelihood of returning to independent living. A shorter interval between the first and second fractures, is associated with worse outcomes in terms of mobility and independent function in the community [20,21].

Despite its clinical importance, the issue of recurrent fractures in post-acute settings has been scarcely studied. Expanding research in this area may enhance communication among interdisciplinary healthcare teams, patients, and caregivers as to rehabilitation expectations, discharge planning, and post-rehabilitation support needs. Moreover, greater awareness of the risk of recurrent fractures may promote appropriate osteoporosis management for all patients admitted to post-acute rehabilitation following a hip fracture.

The objectives of the current study were to compare the demographic, clinical, and functional characteristics of patients who sustained a single hip fracture with those who experienced recurrent fractures, and to identify independent predictive factors associated with the occurrence of subsequent hip fractures. We hypothesized that patients who experience a recurrent fracture following an initial hip fracture will exhibit significantly lower physical and cognitive functional status, measured at the time of the first fracture, compared to those who experience only a single hip fracture.

## 2. Methods

### 2.1. Study Design

This retrospective cohort study was based on data extracted from the electronic health records of Clalit Health Services (CHS), the largest integrated payer-provider healthcare organization in Israel. The study was conducted according to the guidelines of the Declaration of Helsinki and approved by the Institutional Review Board of Beit Rivka Geriatric Rehabilitation Center, Helsinki number: 0475-23, Approval date: 23 October 2023.

### 2.2. Participants

The study population comprised patients aged ≥65 years who were insured by CHS and possessed a documented diagnosis of a hip fracture between 2016 and 2023. Inclusion criteria encompassed all individuals within this age group who sustained a first hip fracture during the study period. No exclusion criteria were applied.

### 2.3. Procedure

Data were extracted from the CHS electronic medical records using the organization’s Research Data Sharing Platform, powered by MDClone (https://www.mdclone.com, accessed on 31 October 2024). CHS provides healthcare services to over 4.5 million individuals, representing ~54% of the Israeli population. Eligible patients were categorized into two groups: (a) those who had sustained a single hip fracture, and (b) those who had experienced a recurrent fracture following an initial hip fracture. The dataset included demographic, clinical, and functional variables.

Demographic variables included age, gender, and socioeconomic status (SES) categorized as low, medium, or high. Both population sector and SES were defined based on the designation of the patient’s primary care clinic, as classified by the Israeli Central Bureau of Statistics. Clinical variables included the type of recurrent fracture (pelvis, vertebrae, hip, upper or distal lower extremities), comorbidities, serum albumin levels at the time of the initial fracture, and the number of medication dispensations during the first year post-fracture. Medications of interest included antidepressants, antipsychotics, hypnotics and sedatives, and osteoporosis treatments.

Functional variables included the Functional Independence Measure (FIM) score, assessed at admission and discharge from the rehabilitation center [22,23,24]. The FIM comprises 18 items across three domains: activities of daily living (ADL) (8 items), mobility (5 items), and cognitive function (5 items), each rated on a 7-point scale. The motor FIM (mFIM) comprises 13 ADL and mobility items. The maximum score for the total FIM is 126 and 91 for the mFIM. Functional gain was calculated as the difference between discharge and admission scores. The patient’s FIM score was assessed at multi-disciplinary team meetings.

To estimate the pre-fracture disability level, data were also collected from the Long-Term Care (LTC) benefit score of the Israeli National Insurance Institute. This benefit is provided to retirees living at home and is based on the level of assistance required for daily functioning. The score is stratified into six levels, ranging from 2.5 to 10.5 points, with higher scores indicating greater functional impairment.

### 2.4. Statistical Analysis

Statistical analyses were conducted using RStudio (version 2022.07.1) [25]. After defining the final sample, all participants with sufficient data were included in the analysis. A secondary data-cleaning step converted a small number of invalid entries into missing values (NA), which were subsequently excluded from the analysis. A few non-significant outliers (z > 3) were retained, as they did not materially affect the results. Descriptive statistics estimated demographic and clinical variables reported as means ± standard deviation, medians, or frequencies, as appropriate. Comparisons between the single and recurrent fracture groups were conducted using independent t-tests for continuous variables and chi-square tests for categorical variables. To compare baseline and functional characteristics across the subgroups of patients with different types of recurrent fractures (hip, pelvis, vertebrae, upper or lower extremities), a log transformation was applied to the data to account for unequal group sizes. Variables that violated the assumption of normality, such as SES and LTC benefit scores, were analyzed using non-parametric tests, specifically the Mann–Whitney U test and the Kruskal–Wallis test, as appropriate.

A multivariate stepwise logistic regression was conducted to identify independent predictors of recurrent fractures. Non-significant variables were excluded during the stepwise process based on the Akaike Information Criterion (AIC), which evaluates the relative quality of regression models [26]. The final regression sample included 4577 participants, comprising only individuals with complete data for all variables entered into the model. To evaluate whether restricting the analysis to participants with full data introduced selection bias, we compared the baseline characteristics of those included in the final model (n = 4577) with those excluded due to incomplete data (n = 35,814). Standardized mean differences (SMDs) were calculated for all variables, with a SMD > 0.20 indicating a potentially meaningful imbalance. Multicollinearity was assessed using the variance inflation factor (VIF), with values >10 indicating high multicollinearity and compromised model validity [27]. The likelihood ratio test (LRT) assessed whether the inclusion of predictors significantly improved model performance [28]. Model fit was also evaluated using McFadden’s R^2^, calculated as: $R^{2}=1-\frac{ln(L)}{ln(L_{0})}$ where *L* is the likelihood of the full model and *L*_0_ is the likelihood of the intercept-only model. Finally, to control for a false discovery rate with multiple significance testing, a Benjamini–Hochberg (BH) correction was employed for each part of the study. Eventually, in both parts of the study, only *p*-values below the corrected threshold of *p* < 0.034 were considered significant. The calculation for a BH correction is: *α* < 0.05/number of comparisons x rank of comparison.

## 3. Results

The study cohort comprised 40,391 CHS-insured patients who had sustained a hip fracture between the years 2016 and 2023. Of these, 23,027 patients (57%) experienced a single hip fracture, whereas, 17,364 patients (43%) suffered a recurrent fracture within the study period. The proportion of males was significantly higher in the single-fracture group (8491; 36.9%) than in the recurrent-fracture group (5079; 29.3%; *p* < 0.001). Table 1 presents the demographic, clinical, and functional characteristics of the two groups. Patients who had sustained a recurrent fracture were statistically younger, more likely to be female, and had higher serum albumin levels at the time of their initial fracture compared to patients with a single hip fracture. In addition, they exhibited a higher rate of an osteoporosis diagnosis following the first fracture and a greater prevalence of cerebrovascular disease (CVA) and depression. This group was also more likely to have been prescribed antidepressants, hypnotics/sedatives, and osteoporosis medications during the year following the initial fracture.

Conversely, patients with recurrent fractures exhibited lower rates of ischemic heart disease, dementia, and antipsychotic medications use. They also had lower mortality rates and required less home assistance, as reflected by their lower LTC benefit scores. Furthermore, these patients demonstrated significantly higher FIM scores at the time of their initial fracture, indicating better physical and cognitive functioning upon entry to rehabilitation.

Table 2 presents a comparison of the demographic and clinical characteristics of patients who had sustained recurrent fractures, categorized by the anatomical site of their second fracture (hip, pelvis, vertebrae, or other). The majority of recurrent fractures were second hip fractures, and this subgroup included the highest proportion of male patients. Patients who had sustained a recurrent vertebrae fracture exhibited the highest rates of osteoporosis diagnosis following the initial fracture, as well as the highest prevalence of depression, and use of hypnotic/sedative and osteoporosis medications. This group also demonstrated the highest LTC scores, indicating greater functional dependency. Patients with a recurrent pelvic fracture were significantly older and exhibited the highest prevalence of respiratory disease.

The final results of the backward stepwise logistic regression model are presented in Table 3. The model was based on a final sample of 4577 participants with complete data for all variables included in the analysis. Variables retained in the final model were selected according to their clinical relevance, statistical significance, and contribution to overall model fit, as reflected by their effect-size estimates. Ischemic heart disease was excluded from the final model because it did not reach statistical significance (*p* > 0.05) and contributed minimally to model performance (AIC = 6032.8). To assess potential selection bias arising from the restriction to participants with complete data, standardized mean differences (SMDs) were calculated between those included in the regression analysis and those excluded. All variables demonstrated minimal imbalance, with the exception of osteoporosis diagnosis at the time of the first fracture (77.3% among included participants vs. 51% among those not included; SMD = 0.51).

The final model demonstrated good fit to the data, as evidenced by a LRT, χ^2^(9) = 49,182.41, *p* < 0.001. Multicollinearity was minimal, with all VIF values at <1.1. The model accounted for 89.1% of the variance in the likelihood of a recurrent fracture, as measured by McFadden’s R^2^. The most influential predictors of a recurrent fracture were a diagnosis of osteoporosis at the time of the first fracture (OR = 2.144, *p* < 0.001), CVA (OR = 1.16, *p* = 0.038), depression (OR = 1.23, *p* = 0.001), and female gender (OR = 0.86, *p* = 0.027; indicating lower odds for males). While age and FIM scores at the time of the first fracture reached statistical significance, their contribution to the model’s predictive strength was modest. Albumin levels and a diagnosis of dementia at the time of the first fracture were not significantly associated with recurrent fracture risk (*p* > 0.05).

## 4. Discussion

In this large-scale, real-world data study (n = 40,391), we compared the demographic, clinical, and functional characteristics of patients who had sustained a recurrent fracture following an initial hip fracture with those who did not. Our findings identified osteoporosis, a prior diagnosis of CVA, depression at the time of the initial hip fracture, and female gender as significant independent predictors of recurrent fractures. Although, increasing age and lower functional status, as measured by the FIM, were also associated with a heightened risk of recurrence, their relative contribution to the predictive model, was modest. Notably, serum albumin levels and a diagnosis of dementia at the time of the initial fracture were not found significantly associated with a recurrent fracture risk in the multivariable analysis.

These findings align with previous research, emphasizing the multifactorial nature of fracture risk in older adults [29]. Osteoporosis is a well-established risk factor for both initial and recurrent fractures, particularly, among elderly women. This elevated risk is attributed to a combination of hormonal, biological, and clinical factors. Postmenopausal women undergo accelerated bone loss as a result of an estrogen deficiency, increasing their susceptibility to fragility fractures. Furthermore, women generally have a greater life expectancy following a hip fracture compared to men, thereby, extending the time period during which a second fracture may occur. Despite robust evidence supporting both pharmacological and non-pharmacological interventions, fracture prevention strategies remain underutilized in this high-risk population. Our findings highlight the importance of early identification of high-risk individuals’ adherence to established osteoporosis treatment guidelines [30], and the implementation of tailored rehabilitation and fall prevention programs.

The identification of CVA as a significant predictor is also consistent with previous studies linking stroke-related impairments, such as hemiparesis, balance disturbances, and gait abnormalities, with an increased risk of falls and subsequent fractures [31,32]. These findings underscore the need to incorporate targeted fall-prevention strategies into rehabilitation protocols for hip fracture patients with a history of stroke.

Depression, another independent predictor identified in our model, has been previously associated with reduced physical activity, poor nutritional intake, and lower adherence to medical and rehabilitation treatment, all of which may contribute to decreased bone density and an increased risk of falls and fractures [33,34]. These findings highlight the importance of addressing depressive symptoms as part of a comprehensive approach to hip fracture care. Multidisciplinary interventions that incorporate psychological support, social engagement, and behavioral health services may play a critical role in improving recovery outcomes and reducing the risk of recurrent fractures. Although the FIM is widely used as a validated measure of assessing functional recovery following a hip fracture [35], our findings suggest that it is not a strong predictor of recurrent fractures.

One possible explanation is that the FIM reflects functional capacity during the early post-acute phase and does not capture other long-term risk factors such as balance instability, bone fragility, and environmental hazards. Therefore, while functional independence is essential for successful rehabilitation outcomes and community reintegration, it does not necessarily equate to a reduced risk of future fractures [36].

Interestingly, younger age at the time of the initial hip fracture was associated with an increased risk of recurrent fracture, although age was not found to be a strong independent predictor in the multivariable model. Notably, patients who sustained an additional fracture demonstrated significantly lower mortality (38.9%) compared to those who had not experienced a recurrent fracture (61%). This finding suggests that individuals with greater physiological reserve and better baseline health status are more likely to survive the initial injury and live long enough to be at risk for a second fracture. Conversely, frailer and older patients may not survive long enough to experience a recurrent event, despite their elevated fall risk [37].

Serum albumin and dementia were not found to be significant predictors in our final model. Although low albumin is commonly considered a marker of frailty, malnutrition, and worse post-fracture outcomes [38], it may not specifically predict recurrence. Similarly, while dementia is a well-established risk factor for falls and fractures [8], its lack of predictive value for recurrence may be attributed to factors such as reduced life expectancy, advanced functional decline, and limited mobility. Additionally, the underdiagnosing or underreporting of non-displaced or vertebral fractures, particularly among cognitively impaired individuals, may further contribute to this finding [39,40].

This study has several limitations. Its retrospective design and reliance on electronic medical records introduces the potential for documentation bias. The fact that the regression analysis was conducted on only a subset of the sample could, in principle, introduce bias into the results. Nonetheless, as no significant differences were identified between the two groups across all variables except one, the probability of meaningful bias arising from this limitation appears to be minimal. Moreover, key variables such as bone mineral density, detailed fall history, environmental hazards, and adherence to prescribed medications were either not systematically recorded or were inconsistently documented within the dataset. These limitations may have affected the depth of our analysis and attenuated the strength of some associations.

## 5. Conclusions

This study underscores the multifactorial nature of risk factors associated with recurrent fractures following an initial hip fracture. Key independent predictors identified include osteoporosis, history of a CVA, depression at the time of the first fracture, and female gender. These findings highlight the importance of implementing long-term, individualized prevention strategies that extend beyond bone health and to also address mental health and functional capacity. Targeted screening and tailored interventions for high-risk patients may reduce the incidence of subsequent fractures. Future prospective research should evaluate the effectiveness of integrated post-fracture care models that incorporate recurrent fracture risk assessment, psychological support, and fall prevention strategies to improve outcomes in this vulnerable population.

## Figures and Tables

**Table 1:** Comparison of baseline characteristics between patients with a single hip fracture and those with recurrent fracture following an initial hip fracture.

Characteristics	Patients with a Single Hip Fracture (n = 23,027)	Patients with a Recurrent Fracture Following an Initial Hip Fracture (n = 17,364)	*^,§^ *p*-Value
**Demographic characteristics**			
Age at first fracture (Mean, SD)	83.0 ± 8.2	80.3 ± 7.9	<0.001
Males (n, %)	8491 (36.9%)	5079 (29.3%)	<0.001
Socio-economic 5-score (Mean, SD)	2.5 ± 1.34	2.6 ± 0.87	0.978
**Clinical characteristics**			
BMI at first fracture (Mean, SD) ^µ^	26.1 ± 5.2	26.5 ± 5.2	<0.001
Albumin level at first fracture (Mean gr%, SD) ^µ^	3.4 ± 0.5	3.5 ± 0.5	<0.001
Osteoporosis diagnosis after the first fracture (n, %)	10,450 (45.4)	12,398 (71.4)	<0.001
Ischemic heart disease (n, %)	6214 (27.0)	4244 (24.4)	<0.001
Congestive heart failure (n, %)	6653 (28.9)	4874 (28.1)	0.072
Diabetes mellitus (n, %)	8619 (37.4)	6558 (37.8)	0.518
Hypertension (n, %)	16,155 (69.1)	12,006 (70.2)	0.029
Cerebrovascular disease (n, %)	3325 (14.4)	3152 (18.2)	<0.001
Respiratory disease (n, %)	3815 (16.6)	2977 (17.1)	0.128
Dementia (n, %)	2355 (10.2)	1637 (9.4)	0.008
Depression (n, %)	8295 (36.0)	7217 (41.6)	<0.001
**Medication dispensation amount**			
Anti-depressants (M, SD)	2.8 ± 5.4	3.7 ± 6.1	<0.001
Anti-psychotics (M, SD)	0.7 ± 2.8	0.5 ± 2.6	<0.001
Hypnotics and sedatives (M, SD)	2.4 ± 4.6	3.1 ± 5.2	<0.001
Osteoporosis medications (M, SD)	0.5 ± 2.0	1.2 ± 2.7	<0.001
**Functional characteristics**			
FIM score at first fracture (M, SD) ^µ^	57.2 ± 26.5	63.6 ± 27.0	<0.001
Deceased (n, %)	14,054 (61.0)	6666 (38.9)	<0.001
Mean age at death (M, SD) ^µ^	86.5 ± 7.9	86.4 ± 8.2	0.514
Insurance aid scores (M, SD) ^µ^	7.5 ± 2.8	6.9 ± 2.7	<0.001

* *p*-value from the ANOVA/chi-square test; ^§^ Significant at *p* < 0.044 (Benjamini–Hochberg correction); ^µ^ Data for these variables was available for only part of the sample: BMI n = 27,756; Albumin level at the first fracture, n = 18,510; FIM score at the first fracture, n = 7304; Mean age at death, n = 20,720; Insurance aid score, n = 17,672; BMI, kg/ht^2^; FIM, Functional Independence Measure.

**Table 2:** Demographic and clinical characteristics of patients with recurrent fracture, stratified by anatomical site of the second fracture.

Anatomic Region of Recurrent Fractures\Characteristics	Pelvis	Vertebrae	Hip	Other ^µ^	* *p*-Value
**Demographic characteristics**					
Frequency (n, % of total)	4312 (17.1)	2558 (10.1)	11,234 (44.5)	7134 (28.3)	<0.001
Male (n, % of group type)	1129 (26.2)	621 (24.3)	3436 (30.6)	1747 (24.5)	<0.001
Mean age (SD) ^§^	80.8 ± 7.7	80.5 ± 7.5	79 ± 7.6	80.1 ± 8.0	<0.001
Socio-economic score	2.5 (1.3)	2.5 (1.3)	2.6 (1.3)	2.5 (1.3)	0.219
**Clinical characteristics**					
BMI (kg/ht^2^, SD) ^§,β^	26.1 ± 4.9	26.1 ± 4.9	26.5 ± 5.1	26.8 ± 5.4	<0.001
Albumin level (gr%, SD) ^§,β^	3.5 ± 0.5	3.5 ± 0.5	3.6 ± 0.5	3.5 ± 0.5	<0.001
Osteoporosis diagnosis (n, %) ^§^	3169 (73.5)	2022 (79.0)	8655 (77.0)	4857 (68.1)	<0.001
Ischemic heart disease (n, %)	1331 (30.9)	787 (30.8)	3099 (27.6)	2015 (28.2)	0.898
Congestive heart failure (n, %)	204 (4.7)	114 (4.5)	533 (4.7)	361 (5.1)	<0.001
Diabetes mellitus (n, %)	71 (1.6)	29 (1.1)	163 (1.5)	116 (1.6)	0.273
Hypertension (n, %)	1600 (37.1)	934 (36.5)	4220 (37.6)	2748 (38.5)	0.039
Cerebrovascular disease (n, %)	841 (19.5)	496 (19.4)	2080 (18.5)	1301 (18.2)	0.271
Respiratory disease (n, %)	3033 (70.3)	1776 (69.4)	7675 (68.3)	4985 (69.9)	<0.001
Dementia (n, %)	410 (9.5)	241 (9.4)	1011 (9.0)	707 (9.9)	0.225
Depression (n, %)	2008 (46.6)	1225 (47.9)	4687 (41.7)	3026 (42.4)	<0.001
**Medication**					
Anti-depressants (M, SD)	4.2 ± 6.4	4.3 ± 6.4	3.7 ± 6.1	3.7 ± 3.7	<0.001
Anti-psychotics (M, SD)	0.46 ± 2.4	0.52 ± 2.7	0.46 ± 2.4	0.5 ± 2.5	0.387
Hypnotics and sedatives (M, SD)	3.52 ± 5.5	3.59 ± 5.5	3.1 ± 5.2	3.1 ± 5.1	<0.001
Osteoporosis medications (M, SD)	1.4 ± 3	1.5 ± 3	1.3 ± 1.4	1.2 ± 2.8	<0.001
**Functional characteristics**					
FIM score at first fracture ^β^	65.15 ± 25.1	64.24 ± 24.7	65.09 ± 27.4	64.13 ± 27	0.074
Deceased (n, %)	1649 (38.2)	964 (37.7)	3891 (34.6)	2873 (40.3)	<0.001
Mean age at death (M, SD) ^β^	86.47 ± 7.4	86.12 ± 7.3	86.42 ± 7.5	86.21 ± 7.8	0.417
Insurance aid scores (M, SD) ^β^	7.07 ± 2.6	7.07 ± 2.6	6.8 ± 2.7	7.01 ± 2.7	0.004

* *p*-value from the ANOVA/chi-square test; ^§^ documented at the first fracture; ^µ^ Other; upper and distal lower limb bone fractures; ^β^ Data for these variables was available for only part of the sample: BMI n = 14,968; Albumin level at the first fracture, n = 8561; FIM score at the first fracture, n = 3487; Mean age at death, n = 6666; Insurance aid score, n = 10,248; BMI, kg/ht^2^; FIM, Functional Independence Measure.

**Table 3:** Multivariable logistic regression results of predicting factors for recurrent fractures.

Predictors	Odds Ratio	Β	z-Value [95% CI]	*p*-Value
(Intercept)	6.326	1.845	4.21	<0.001
Age *	0.960	−0.040	−9.71	<0.001
Gender (Male)	0.861	−0.149	−2.206	0.027
Albumin Level *	1.132	0.124	1.96	0.051
Cerebrovascular Disease	1.163	0.151	2.07	0.038
Osteoporosis *	2.144	0.763	10.10	<0.001
Depression	1.232	0.209	0.33	<0.001
Dementia	0.870	−0.140	−1.63	0.102
FIM scores	1.01	0.007	6.20	<0.001

* Values and diagnoses at first fracture; FIM, Functional Independence Measure.

## Data Availability

The original contributions presented in this study are included in the article. Further inquiries can be directed to the corresponding author.

## Abbreviations

CHS	Clalit Health Services
SES	Socioeconomic status
FIM	Functional Independence Measure
ADL	Activities of daily living
mFIM	Motor FIM
LTC	Long-Term Care
AIC	Akaike Information Criterion
VIF	Variance inflation factor
BH	Benjamini–Hochberg
CVA	Cerebrovascular disease
